# Intrauterine infusion of autologous platelet-rich plasma modulates endometrial immune status and improves pregnancy outcomes in patients with persistent chronic endometritis

**DOI:** 10.3389/fimmu.2025.1528522

**Published:** 2025-05-15

**Authors:** Xian Chen, Mingye Chen, Meng Liu, Lingbin Qi, Zhiqiang Liu, Cong Chen, Binfa Liang, Xiaobing Yang, Tao Zhang, Yuye Li, Ruochun Lian

**Affiliations:** ^1^ Shenzhen Key Laboratory of Reproductive Immunology for Peri-implantation, Shenzhen Zhongshan Institute for Reproductive Medicine and Genetics, Shenzhen Zhongshan Obstetrics and Gynecology Hospital, Shenzhen, China; ^2^ Guangdong Engineering Technology Research Center of Reproductive Immunology for Peri-implantation, Shenzhen, China; ^3^ Reproductive Medical Center, Department of Gynecology and Obstetrics, Tongji Hospital, Tongji University School of Medicine, Shanghai, China; ^4^ Guangdong Medical University, Dongguan Key Laboratory of Medical Bioactive Molecular Developmental and Translational Research, Guangdong Provincial Key Laboratory of Medical Immunology and Molecular Diagnostics, Dongguan, China; ^5^ Department of Obstetrics and Gynecology, Faculty of Medicine, The Chinese University of Hong Kong, Hong Kong, Hong Kong SAR, China

**Keywords:** chronic endometritis, platelet-rich plasma, pregnancy outcomes, endometrial immune cells, infertility

## Abstract

**Background:**

Chronic endometritis (CE) has been widely recognized as a potential cause of infertility, however, access to effective treatment is a formidable challenge due to the rudimentary understanding of the pathogenesis of persistent CE. Here, we aimed to analyze the impact of platelet-rich plasma (PRP) treatment on pregnancy outcomes and the endometrial microenvironment in patients with persistent CE.

**Methods:**

A total of 89 infertility patients were selected, including 56 non-CE (as the control group) and 33 persistent CE. The persistent CE patients received an intrauterine infusion of PRP four times before embryo transfer. Immunohistochemistry staining and transcriptomic sequencing were used to investigate the uterine-specific role of PRP in patients with persistent CE.

**Results:**

The implantation rate and clinical pregnancy rate were significantly increased in the cured CE group compared to the non-cured CE group. After PRP treatment, the proportions of endometrial CD8^+^ T cells, CD56^+^ NK cells, Foxp3^+^ Treg cells, and T-bet^+^ Th1 cells were significantly decreased in patients with persistent CE. Specifically, DEG analysis showed that genes implicated in endometrial receptivity-related and antimicrobial were upregulated and genes involved in the immune response processes were downregulated in cured CE patients after PRP treatment. Functional enrichment analysis suggested that the effects of changes in leukocyte chemotaxis-related genes played a critical role in the endometrial immune environment.

**Conclusions:**

Autologous PRP treatment has been shown as a potentially successful therapy for improving pregnancy outcomes by reconstructing the uterine local immune microenvironment to improve endometrial receptivity in patients with persistent CE.

## Introduction

Chronic endometritis (CE) is a disease of persistent inflammation of the endometrium, characterized by abnormal infiltration of endometrial stromal plasma cells (1). It is frequently associated with infertility as it may reduce endometrial receptivity (2, 3). Numerous studies have shown that the most common causes of CE arise from infection with pathogenic micro-organisms, such as *Escherichia coli*, *Streptococcus* spp., *Staphylococcus* spp., *Chlamydia*, and some viruses (4, 5). Thus, oral antimicrobial regimes are considered to be the gold standard in the treatment of CE. It has been reported that antimicrobial agents eliminate endometrial stromal plasmacytes, but the endometrial or intrauterine microbial profile alterations of patients with CE remain unclear (6). Clinical evidence has confirmed that certain persistent CE patients do not respond satisfactorily to a wide spectrum of antibiotic treatments such as doxycycline, ciprofloxacin, ofloxacin, amoxicillin, josamycin, metronidazole, clavulanate, and minocycline, which are closely associated with pregnancy failure (7). Thus, there is a crucial need for specific and effective therapies to improve persistent CE patient outcomes.

Platelet-rich plasma (PRP) is a promising therapeutic modality in various medical cases, including osteoarthritis, ovarian dysfunction, and endometrial disorders. It has been introduced due to its antimicrobial and anti-inflammatory properties (8, 9). PRP is prepared by peripheral blood withdrawal following centrifugation to achieve a high concentration of platelets. Besides, PRP contains numerous growth factors such as platelet-derived growth factor (PDGF), epidermal growth factor (EGF), transforming growth factor-β (TGF-β), vascular endothelial growth factor (VEGF), and other cytokines, which exhibit a vital role in various biological processes, including cell proliferation, differentiation, angiogenesis, immunomodulation, regulation of and apoptosis (10–12). Such attributes underscore their potential application in assisted reproductive technology (ART), where they are believed to contribute to tissue repair and regeneration (10). In recent decades, valuable insights have been gained into the efficacy and safety of PRP in ART in a complex clinical situation characterized by limited treatment options (8). While PRP has been investigated for both ovarian rejuvenation and endometrial enhancement in ART, a recent randomized controlled trial has failed to demonstrate significant improvements in ovarian reserve or response (13). Of particular clinical relevance, current evidence suggests PRP may offer greater potential for endometrial augmentation, particularly in cases of thin or compromised endometrium. Currently, clinical studies have demonstrated the effectiveness of PRP combined with antibiotic treatments in improving the live birth rate and clinical pregnancy rate for RIF patients with CE during IVF-ET (14). Nevertheless, except for one case report (6), there is a lack of existing literature on the effectiveness of PRP monotherapy for patients with persistent CE, and the cellular and molecular mechanisms of PRP treatment for this condition are still unclear.

In this study, our first objective was to evaluate the efficacy of autologous PRP treatment in patients with persistent CE. We then analyzed the pregnancy outcomes of freeze-thaw embryo transfer (FET) in these patients. Additionally, considering the immunomodulatory properties of PRP, we aimed to investigate its effect on endometrial immune cells in patients with persistent CE. Furthermore, to understand the uterine-specific role of PRP in patients with persistent CE, we conducted gene expression profiling of human mid-secretory endometrium from patients with persistent CE before and after PRP treatment using RNA sequencing (RNA-seq).

## Materials and methods

### Subjects

This retrospective cohort study of infertile patients with accepted indications for IVF-ET who attended the Fertility Centers of Shenzhen Zhongshan Urology Hospital was conducted. All patients included in this study had tubal disorders or unexplained factors at their first IVF treatment. The period of recruitment of participants was from May 2022 to April 2024. The study was approved by the Investigation and Ethics Committee of the Shenzhen Zhongshan Obstetrics and Gynecology Hospital (Approval No. SZZSECHU-2022013). Informed written consent was obtained from each patient before the endometrial biopsy. The inclusion criteria were: 1) age < 40 years; 2) regular menstrual cycling; 3) normal karyotypes; 4) negative serological tests for human immunodeficiency virus, syphilis, hepatitis B virus, and hepatitis C virus; 5) normal basal levels of hormones, including follicle-stimulating hormone (FSH), luteinizing hormone (LH), estradiol (E2) or progesterone (P), which were measured on the third day of the menstrual period; 6) normal uterine anatomical; 7) endometrial tissue biopsies were obtained during the mid-luteal phase of the menstrual cycle; 8) had a successful ovarian stimulation and subsequent embryo transfer. To reduce the study bias, we excluded factors that may affect the morphology and function of the endometrium and pregnancy outcomes. Thus, patients were excluded from the study if they: 1) had endometriosis, polycystic ovary syndrome, adenomyosis, or leiomyoma; 2) couples with male infertility; 3) had autoimmune diseases (including antiphospholipid syndrome, systemic lupus erythematosus, autoimmune thyroid disease, Sjogren syndrome, etc). All the patients’ information, including their age, body mass index (BMI), basal hormone levels, infertility type, number of embryos transferred, transferred embryo type, embryo quality, and previous pregnancy outcomes, were collected from the central database of our hospital on July 1, 2024. No one was pregnant when the endometrium samples were collected during the mid-luteal phase.

### Endometrial biopsy and diagnosis of chronic endometritis

All endometrial biopsies were taken by using an endometrial curette (Gynetics, Lommel, Belgium) during the mid-luteal phase (LH days 7-9). The specimens were incubated overnight with 10% neutral buffered formalin at room temperature and then embedded in paraffin wax. All endometrial samples from patients before and after PRP treatment were used to detect endometrial immune cells by immunohistochemistry. Among 20 participants with cured CE after PRP treatment, three patients’ endometria were carried on RNA-seq before and after PRP treatment.

The diagnosis of CE was based on the author’s previous study (2), which was defined as more than three high-power fields (HPF; magnification ×200) with five or more CD138^+^ plasma cells per HPF. The diagnosis of persistent CE was based on three or more separate menstrual cycles of consecutive CD138^+^ plasma cells at the mid-luteal phase. Fewer than five CD138^+^ or no plasma cells per HPF in each of the 30 randomly selected HPFs indicated the absence of CE (non-CE).

### Immunohistochemistry staining and image analysis

Endometrial tissues were fixed with 10% neutral buffered formalin overnight, and then dehydrated and embedded in paraffin. Paraffin sections (4 μm) were prepared, dewaxed, hydrated, and the endogenous peroxides were quenched with 3% H_2_O_2_. After heat-mediated antigen retrieval, the slides were incubated with monoclonal antibodies presented in **Supplementary Table 1**. After incubation with prediluted HRP-conjugated secondary antibodies (Typing, China), the sections were exposed to DAB and counterstained with hematoxylin. All immunohistochemistry staining was performed on a Leica Bond III automated immunostainer (Leica Microsystems, Bannockburn, IL).

Quantitative analysis of endometrial immune cells was performed using an Olympus SLIDEVIEW VS200 system (Olympus, Tokyo, Japan). First, the slides were scanned at lower magnification, and then images of 30 random images per section were captured in high-power fields (HPFs; magnification ×200). All immune cell populations from each panel were characterized and quantified with the use of the cell segmentation and phenotype cell tool of the HALO Analysis software (Indica Labs, Corrales, NM, USA) under the supervision of the same pathologist. The concentration of each immune cell population was assessed as a percentage of all endometrial cells in each of 30 randomly selected HPFs (magnification ×200).

### Treatment

In this study, patients with CE were treated with antibiotics starting from the first day of menstruation, which included 100 mg of doxycycline hydrochloride orally twice daily and 400 mg of metronidazole orally once daily for 14 days. To observe the effect of treatment, the endometrium was collected again after 7 days of antibiotic treatment (during the next mid-luteal stage) for immunohistochemical staining of CD138^+^ cells. Patients treated successfully (fewer than five CD138^+^ or no plasma cells per HPF in each of 30 randomly selected HPFs) for CE after one cycle of antibiotic treatment were eligible for subsequent FET. If the outcomes showed that patients were still positive for CD138, they would receive a second course of antibiotic treatment with 500 mg of levofloxacin orally twice daily and 400 mg of metronidazole (Gold Day Pharmaceutical Co.) orally once daily for 14 days (15). If patients consistently had three or more cycles of CD138^+^ plasma cells in the endometrium, they would be administered PRP treatment in the subsequent menstrual cycle after antibiotic treatment. The endometrium was retaken during the mid-luteal stage.

The PRP was prepared from autologous blood using a 2-step centrifugation by a modified method (16). Briefly, 8.5 mL of peripheral blood was drawn into ACD-A tubes and centrifuged at 190 g for 10 min. The buffy coat layer and the plasma layer were collected and transferred to a new tube to be centrifuged again at 800 g for 15 min. Finally, 1.0 mL of PRP with a good concentration (4~8 times baseline peripheral blood levels) was obtained and stored at 4°C for 1 h or less until infusion. Autologous PRP was infused in the uterine cavity with an intrauterine insemination catheter on the 8–9 days of the menstrual cycle and the process was repeated four times for 12 days, once every 3 days. Patients who were recruited to the control group did not receive any antibiotic or PRP treatment.

### Endometrial preparation and embryo transfer protocol

Patients who received a FET cycle were treated with GnRH agonist and hormone replacement therapy (HRT) before embryo transfer. In Brief, 3.75 mg of Leuprorelin Acetate Microspheres (Ipsen, France) was injected during the mid-luteal phase of the menstrual cycle, 29 days after initiating the hormone replacement protocol as in HRT. When the endometrial thickness reached 7 mm, 40 mg of oral dydrogesterone (Duphaston; Abbott, Netherlands) and 90 mg of vaginal progesterone (Crinone; Merck, Germany) were taken daily for luteal support, after 4 or 6 days, the cleavage-stage embryos or blastocysts were transferred.

Embryos were vitrified and thawed by using the conventional method. The available blastocysts were defined as high-quality (AA, AB, BA, BB) or medium-quality (AC, CA, BC, CB) blastocysts (17), used for transfer on day 5 after fertilization under the guidance of ultrasound. Cleavage embryos were transferred into the uterus 3 days after oocyte retrieval. A serum HCG assay was performed on day 11 (if blastocyst was transferred) or day 13 (if cleavage embryo was transferred) after the embryo was transferred, and continued until 12 weeks of gestation for pregnant patients (4).

### Pregnancy outcome measures

The outcome measures assessed were implantation rate, β-hCG positive rate, clinical pregnancy rate, and miscarriage rate. The clinical pregnancy rate was defined as the observation of a gestational sac on ultrasound 4–5 weeks after embryo transfer. The implantation rate was defined as the number of gestational sacs observed on ultrasound scanning divided by the number of embryos transferred. A miscarriage was defined as a clinical pregnancy that was lost before 20 weeks of gestational.

### RNA-sequencing and gene expression quantification

NEBNext^®^ UltraTM RNA Library Kit for Illumina^®^ (NEB, USA) was used to examine the whole-genome expression profiles of three patients before and after PRP treatment. Total RNA was isolated using Trizol reagent according to the manufacturer’s procedure (Invitrogen, Carlsbad, CA, USA). Poly (A) RNA is purified from 1μg total RNA using Dynabeads Oligo (dT)25-61005 (Thermo Fisher, CA, USA) using two rounds of purification. Then the poly(A) RNA was fragmented into small pieces using a Magnesium RNA Fragmentation Module (NEB, cat. e6150, USA). The cleaved RNA fragments were reverse-transcribed to create the cDNA by SuperScript™ II Reverse Transcriptase (Invitrogen, cat. 1896649, USA). An A-base is then added to the blunt ends of each strand, preparing them for ligation to the indexed adapters. Each adapter contains a T-base overhang for ligating the adapter to the A-tailed fragmented DNA. After the heat-labile UDG enzyme (NEB, cat.m0280, USA) treatment of the U-labeled second-stranded DNAs, the ligated products were then amplified to sequence on the Novaseq 6000 platform. The average insert size for the final cDNA library was 300 ± 50 bp.

All sequencing data were Trim Galore (v2.8) to remove the primers and low-quality bases with default parameters. The trimmed reads were aligned to the GRCh38 reference genome with STAR software (v2.7.5c) with the default settings. After mapping, raw counts were achieved by using featureCounts software (v2.0.1). Then, the different expression genes were analyzed using the DESeq2 R package (v1.44.0). Gene ontology analysis was analyzed via ClusterProfiler R package (v4.12.2). The results of all analyses are visualized with ggplot2 (v3.5.1).

### Statistical analysis

All statistical analyses and graphical representations were performed using SPSS 26.0 (IBM Corp., USA) and GraphPad Prism 6 (GraphPad Software, Inc., USA). The Kolmogorov-Smirnov test was used to examine the distribution of continuous variables. The continuous variables with normal distribution were shown as mean ± standard deviation (mean ± SD) and analyzed by the Wilcoxon rank-sum test. The median (interquartile range) [M (P25, P75)] has been reported for continuous variables with a non-normal distribution. The categorical variables were shown as numbers and percentages and were analyzed using chi-square or Fisher’s exact tests. The paired-sample test was used to compare the differences between before and after PRP treatment data. For normally distributed variables, paired t-tests were utilized, while for non-normally distributed variables, the Wilcoxon rank-sum test was applied to the paired samples. In all comparisons, a two-tailed *P* values < 0.05 were considered statistically significant.

## Results

**Figure 1:**
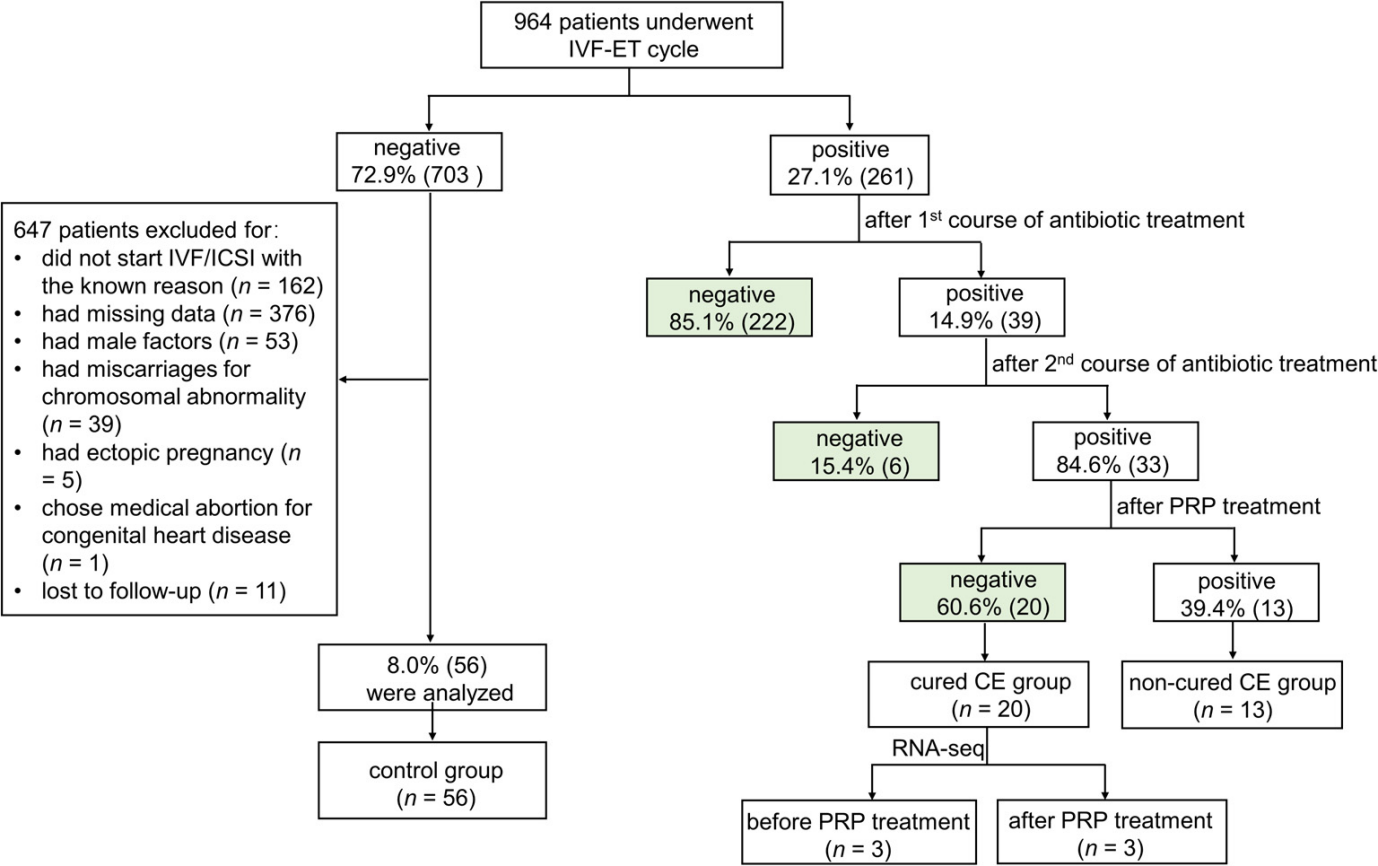
The clinical treatment procedure of persistent CE patients.

In this study, 964 patients who underwent IVF-ET cycles were enrolled between May 2022 and April 2024 (**Figure 1**). 27.1% (261/964) of patients were confirmed with CE by immunohistochemical staining of CD138^+^ plasma cells. After treatment with antibiotics, the endometrium of 14.9% (39/261) of persistent positive patients was taken again at the next luteal stage for immunohistochemical detection of CD138^+^ plasma cells. The results showed that 84.6% (33/39) of patients remained CE persistently positive, and 15.4% (6/39) of them became CE negative after treatment. Finally, 33 persistent CE patients have received PRP treatment. 60.6% (20/33) of patients with persistent CE converted to negative after treatment, and 39.4% (13/33) remained CE persistently positive.

### Baseline clinical characteristics

The baseline clinical characteristics of patients are shown in **Table 1**. The number of previous failed embryo transfer cycles was significantly higher in the PRP group than in the control group (*P* < 0.001). No significant differences in age, body mass index (BMI), level of basal hormones, infertility duration, causes of infertility, number of gravidity and parity were observed between the two groups (*P* > 0.05 each).

**Table 1:** The baseline characteristics of the control group and PRP group.

Variables	Control group (*n* = 56)	PRP group (*n* = 33)	*P*
Age (years)	33.5 (31.0, 36.3)	36.0 (33.0, 38.0)	0.075
BMI (kg/m^2^)	21.5 (20.0, 23.7)	21.8 (19.7, 23.6)	0.273
Basal FSH (mIU/mL)	6.4 (5.4, 7.6)	6.8 (5.3, 9.3)	0.699
Basal LH (mIU/mL)	4.6 (3.6, 6.3)	4.7 (3.5, 6.5)	0.554
Basal E2 (pg/mL)	36.4 (29.6, 57.7)	51.3 (37.1, 80.3)	0.082
Basal P (ng/mL)	0.3 (0.2, 0.4)	0.3 (0.2, 0.5)	0.913
Infertility duration (years)	4.0 (3.0, 6.0)	4.0 (3.0, 6.0)	0.397
Cause of Infertility, *n* (%)			0.811
Tubal factor	24 (42.9%)	15 (45.5%)	
Unexplained	32 (57.1%)	18 (54.5%)	
No. of previous failed embryo transfer cycles	0.0 (0.0, 0.0)	1.0 (1.0, 2.0)	<0.001
Gravidity	0.0 (0.0, 0.0)	0.0 (0.0, 0.0)	0.079
Parity	0.0 (0.0, 0.0)	0.0 (0.0, 0.0)	1.000

BMI, body mass index; FSH, follicle-stimulating hormone; LH, luteinizing hormone; E2, estradiol; P, progesterone; PRP, platelet-rich plasma; *P* < 0.05: PRP group versus control group.

### Comparison of CD138^+^ plasma cells before and after PRP treatment in patients with persistent CE

**Figure 2:**
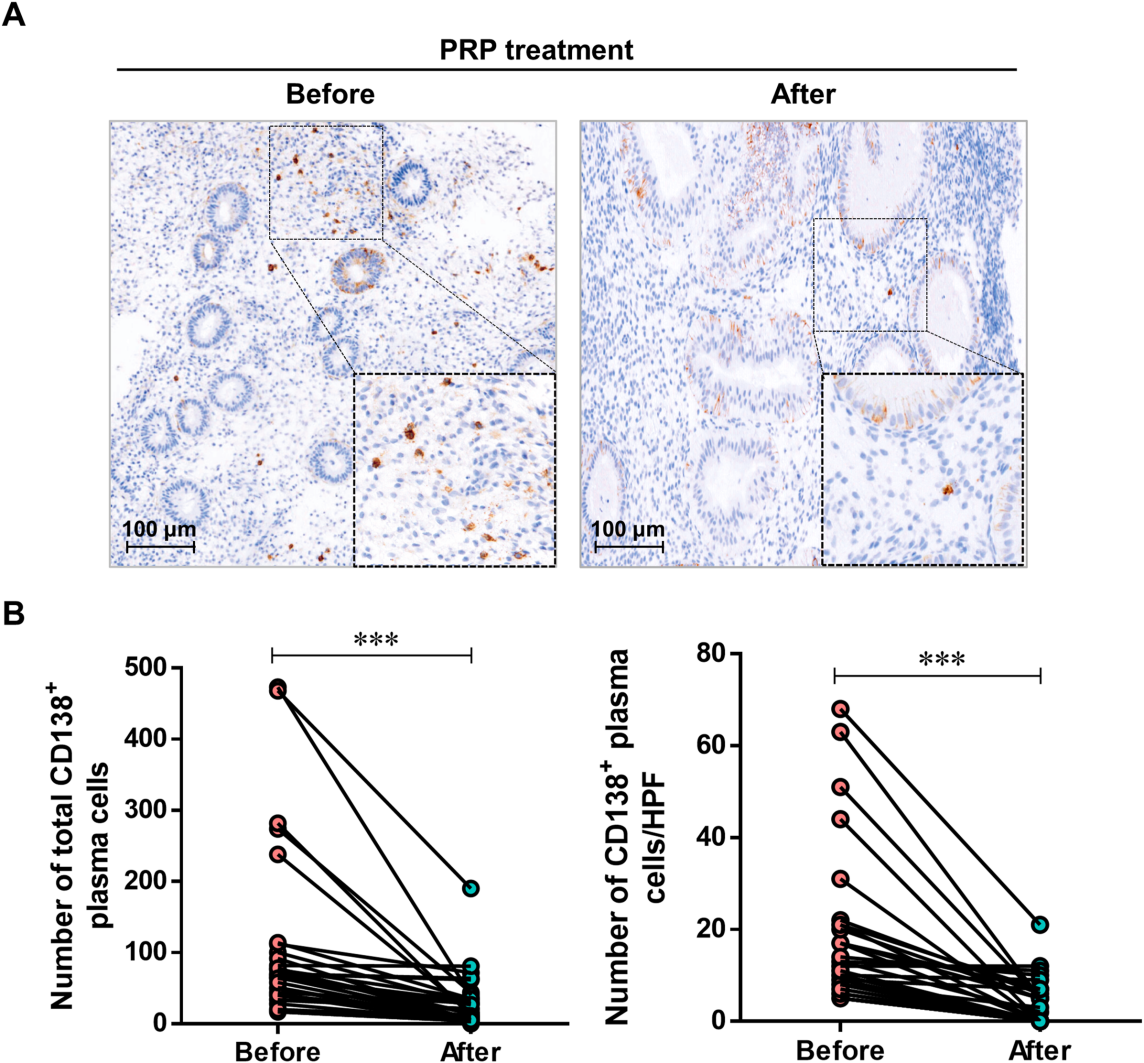
The effect of PRP treatment on endometrial CD138^+^ plasma cells. **(A)** immunohistochemical staining for CD138^+^ plasma cells in persistent CE patients (*n* = 33) before and after PRP treatment. The representative image was taken at a magnification of 200 × field of endometrial cells; Scale bar,100 µm. **(B)** Quantitative analyses of the number of total CD138^+^ plasma cells identified across 30 randomly selected HPFs per endometrial tissue section and the number of CD138^+^ plasma cells per HPF in the endometrial stroma of persistent CE patients before and after PRP treatment. ***P < 0.001.

To investigate the effect of PRP on CD138^+^ plasma cells in patients with consistent CE, the endometrium of 33 patients with persistent CE was retaken at the mid-luteal stage for immunohistochemical detection of CD138^+^ plasma cells after PRP treatment. As shown in **Figure 2**, the number of total CD138^+^ plasma cells (27.6 ± 6.3 *vs.* 103.4 ± 19.9, *P* < 0.001) and the number of CD138^+^ plasma cells per HPF (3.5 ± 0.9 vs. 17.7 ± 2.8, *P* < 0.001) significantly decreased after PRP treatment compared with before treatment, respectively.

### Effect of PRP treatment on pregnancy outcomes in patients with persistent CE

In **Table 2**, the analysis results showed that there were no statistical differences in the endometrial thickness on the day of ET, the number of transferred embryos, embryo type, and high-quality embryos between the control group and the PRP group (*P* > 0.05 each). For the pregnancy outcomes, the positive rate of β-hCG, clinical pregnancy rate, implantation rate, and miscarriage rate were not markedly different between the two groups (*P* > 0.05 each). Of the 33 patients with PRP treatment, 20 were cured of CE, and 13 were not cured of CE. Next, when comparing the pregnancy outcomes of patients between the cured CE and non-cured CE groups after PRP treatment, we noted that the implantation rate (54.3% *vs.* 21.1%, *P* = 0.018) and clinical pregnancy rate (75.0% *vs.* 38.5%, *P* = 0.038) were significantly increased in the cured CE group (**Table 3**). However, there were no significant differences in the positive rate of β-hCG and miscarriage rate between the two groups (*P* > 0.05 each) (**Table 3**).

**Table 2:** Comparison of the pregnancy outcomes between the control group and the PRP group.

Variables	Control group (*n* = 56)	PRP group (*n* = 33)	*P*
Endometrial thickness (cm)	9.0 (8.0, 10.0)	9.0 (7.5, 10.0)	0.225
No. of embryos transferred	2.0 (1.0, 2.0)	2.0 (1.0, 2.0)	0.817
Embryo type, % (*n*/n)			0.164
cleavage	12.5% (7/56)	24.2% (8/33)	
blastocyst	87.5% (49/56)	75.8% (25/33)	
High-quality embryos	1.0 (0.0, 1.0)	1.0 (1.0, 2.0)	0.113
β-hCG positive rate, % (*n*/*n*)	69.6% (39/56)	72.7% (24/33)	0.757
Clinical pregnancy rate, % (*n*/*n*)	62.5% (35/56)	57.6% (19/33)	0.646
Implantation rate, % (*n*/*n*)	40.9% (38/93)	42.6% (23/54)	0.837
Miscarriage rate, % (*n*/*n*)	8.6% (3/35)	5.3% (1/19)	1.000

PRP, platelet-rich plasma; β-hCG, human chorionic gonadotropin-β.

**Table 3:** Comparison of the pregnancy outcomes between the patients with cured and non-cured CE groups after PRP treatment.

Variables	Cured CE group (*n* = 20)	Non-cured CE group (*n* = 13)	*P*
Age (years)	34.5 (31.0, 38.0)	37.0 (35.0, 40.0)	0.080
Endometrial thickness (cm)	9.0 (7.3, 10.0)	9.0 (7.5, 10.0)	0.986
No. of embryos transferred	2.0 (1.3, 2.0)	2.0 (1.0, 2.0)	0.169
Embryo type, % (*n*/n)			0.710
cleavage	45.0% (9/20)	38.5% (5/13)	
blastocyst	55.0% (11/20)	61.5% (8/13)	
High-quality embryos	1.0 (1.0, 2.0)	1.0 (0.0, 1.5)	0.353
β-hCG positive rate, % (*n*/*n*)	85.0% (17/20)	53.8% (7/13)	0.060
Clinical pregnancy rate, % (*n*/*n*)	75.0% (15/20)	38.5% (4/13)	0.038
Implantation rate, % (*n*/*n*)	54.3% (19/35)	21.1% (4/19)	0.018
Miscarriage rate, % (*n*/*n*)	0% (0/15)	25.0% (1/4)	0.211

CE, chronic endometritis; PRP, platelet-rich plasma; β-hCG, human chorionic gonadotropin-β.

### Changes of endometrial immune cells in patients with persistent CE after PRP treatment

**Figure 3:**
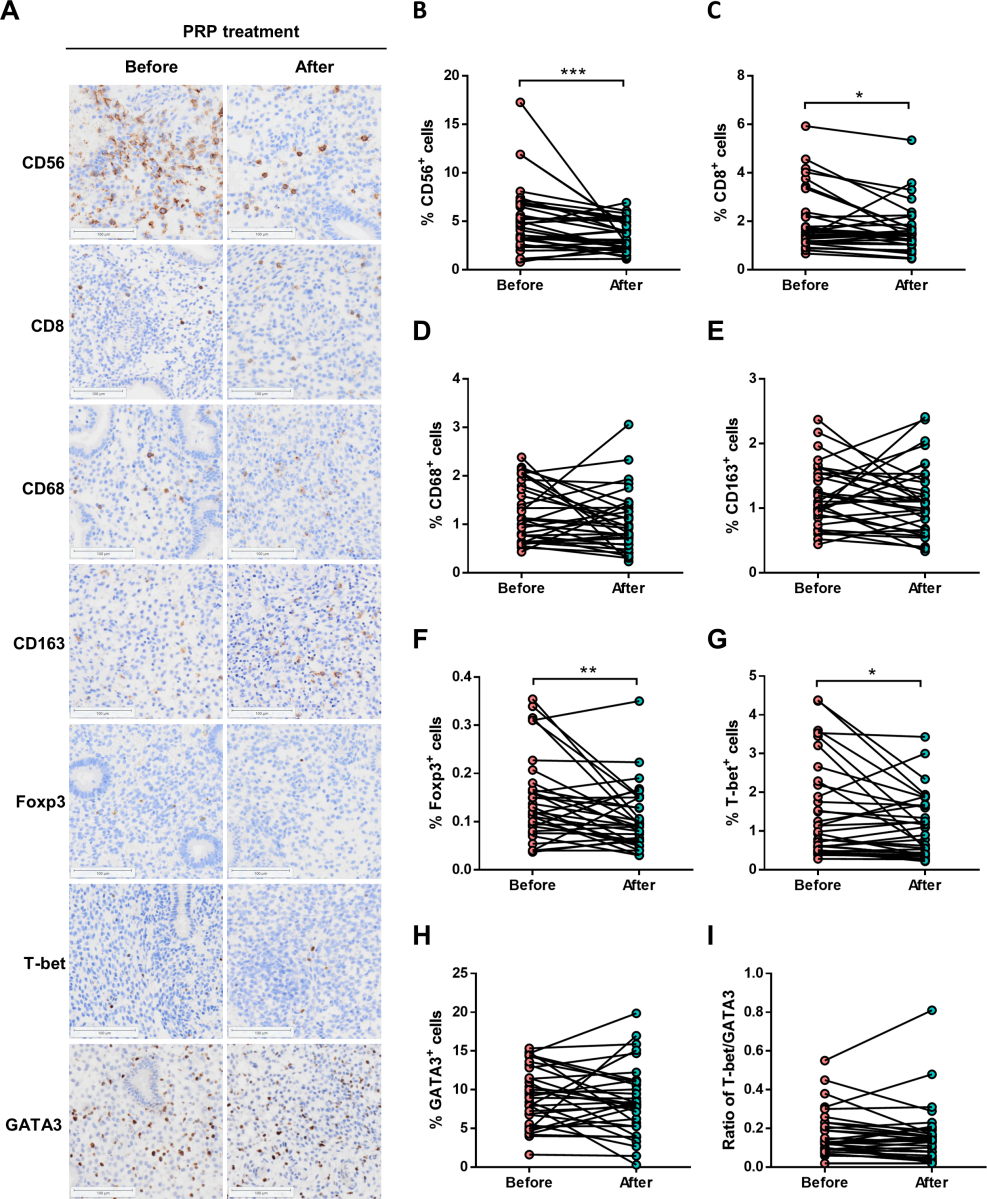
Endometrial immune cells in persistent CE patients before and after PRP treatment. **(A)** immunostaining of endometrial CD56^+^ NK cells, CD8^+^ T cells, CD68^+^ macrophages, CD163^+^ macrophages, Foxp3^+^ Treg cells, T-bet^+^ Th1 cells, and GATA3^+^ Th2 cells in endometrial biopsies from persistent CE patients before and after PRP treatment. The representative image was taken at a magnification of 200 ×field of endometrial cells; Scale bar,100 µm. **(B-I)** Quantitative analyses of percentages on all endometrial cells of uterine cells were performed by using the HALO Analysis system. CE, chronic endometritis; PRP, platelet-rich plasma; *P < 0.05, **P < 0.01, ***P < 0.001.

To evaluate whether the levels of endometrial immune cells were modulated by PRP treatment, the CD56^+^ NK cells, CD8^+^ T cells, CD68^+^ macrophage cells, CD163^+^ macrophage cells, Foxp3^+^ Treg cells, T-bet^+^ Th1 cells, and GATA3^+^ Th2 cells were examined with the use of IHC staining in persistent CE patients before and after PRP treatment (**Figure 3A**). We found that the proportions of endometrial CD56^+^ NK cells (3.5 ± 1.6% *vs.* 5.1 ± 3.4%, *P* < 0.001) (**Figure 3B**), CD8^+^ T cells (1.6 ± 1.0% *vs.* 2.0 ± 1.3%, *P* = 0.019) (**Figure 3C**), Foxp3^+^ Treg cells (0.1 ± 0.1% vs. 0.2 ± 0.1%, *P* = 0.006) (**Figure 3F**), and T-bet^+^ Th1 cells (1.1 ± 0.8% *vs.* 1.5 ± 1.2%, *P* = 0.015) (**Figure 3G**) were significantly decreased in patients with persistent CE after PRP treatment. However, there were no significant differences in the CD68^+^ macrophage cells (1.1 ± 0.6% *vs.* 1.2 ± 0.6%, *P* = 0.261) (**Figure 3D**), CD163^+^ macrophage cells (1.1 ± 0.5% *vs.* 1.2 ± 0.5%, *P* = 0.599) (**Figure 3E**), GATA3^+^ Th2 cells (8.5 ± 4.5% *vs.* 9.0 ± 3.8%, *P* = 0.500) (**Figure 3H**), and the ratio of T-bet^+/^GATA3^+^ (0.2 ± 0.2% *vs.* 0.2 ± 0.1%, *P* = 0.473) (**Figure 3I**) between the before and after PRP treatment groups.

**Figure 4:**
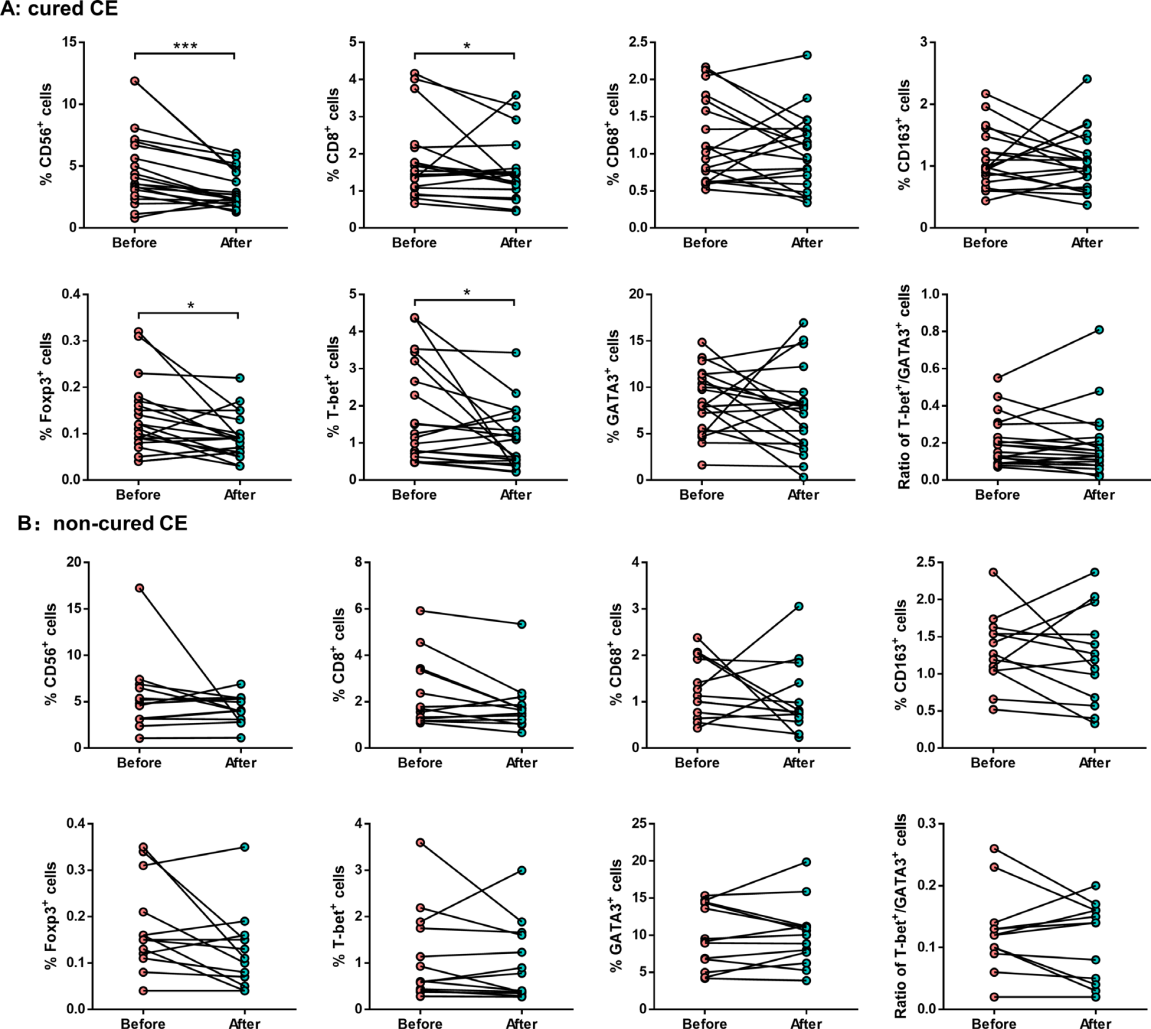
The endometrial immunological alteration between cured CE and non-cured patients before and after PRP treatment. Quantitative analysis shows the proportions of endometrial CD56^+^ NK cells, CD8^+^ T cells, CD68^+^ macrophages, CD163^+^ macrophages, Foxp3^+^ Treg cells, T-bet^+^ Th1 cells, and GATA3^+^ Th2 cells in cured CE patients **(A)** and non-cure CE patients **(B)** before and after PRP treatment. *P < 0.05, ***P < 0.001.

Interestingly, the proportions of endometrial CD56^+^ NK cells (3.1 ± 1.5% *vs.* 4.9 ± 3.1%, *P* = 0.002), CD8^+^ T cells (1.5 ± 0.9% *vs.* 1.9 ± 1.0%, *P* = 0.048), Foxp3^+^ Treg cells (0.1 ± 0.1% *vs.* 0.1 ± 0.1%, *P* = 0.013), and T-bet^+^ Th1 cells (1.1 ± 0.8% *vs.* 1.8 ± 1.4%, *P* = 0.040) were significantly decreased in cured CE patients after PRP treatment (**Figure 4A**). However, no significant differences in non-cured CE patients were observed (**Figure 4B**).

### Endometrial transcriptome suggests that PRP treatment effectively improves the endometrium microenvironment in patients with persistent CE

**Figure 5:**
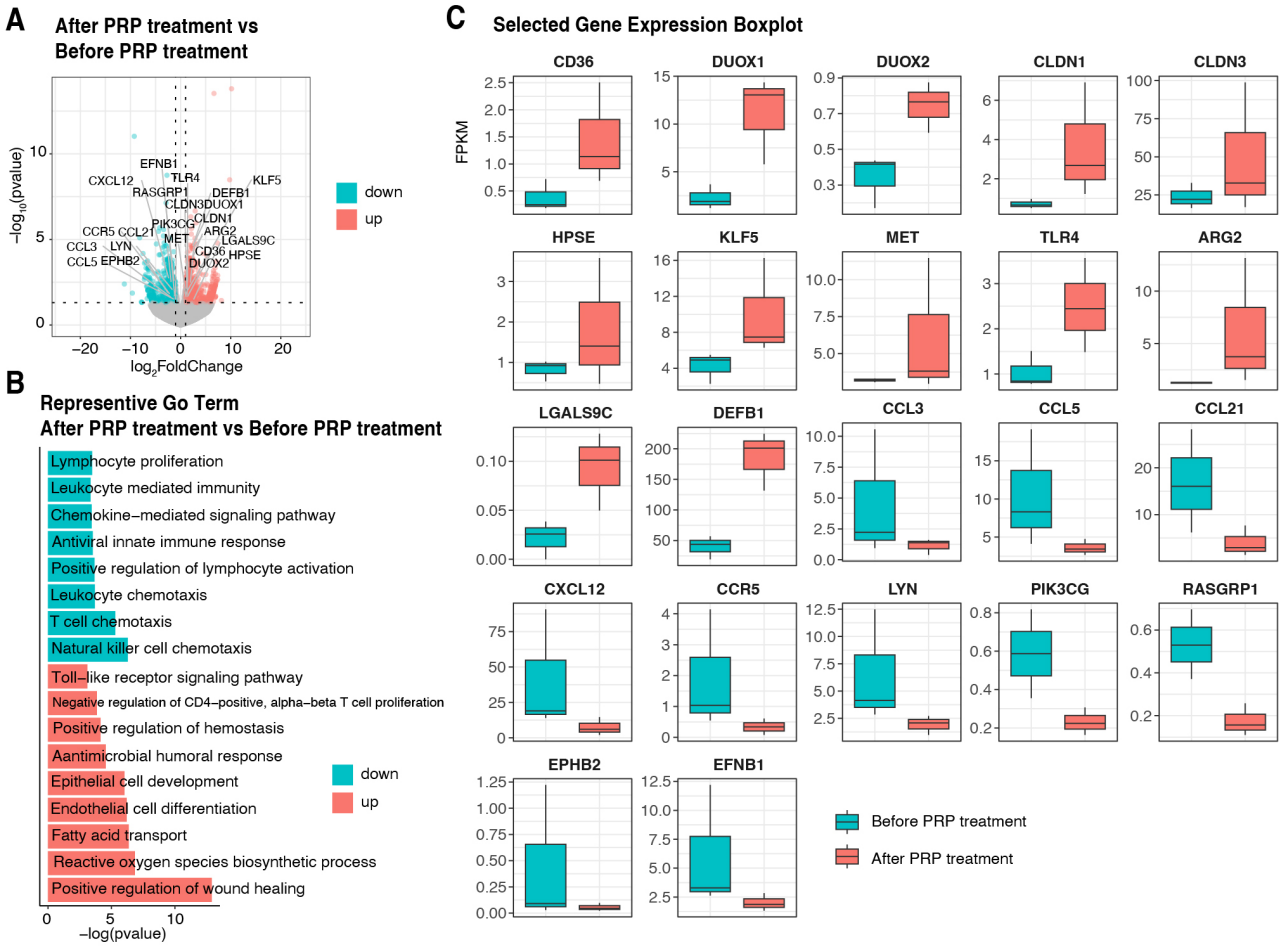
Transcriptome analysis of endometrium in persistent CE patients after PRP treatment using RNA-sequencing. **(A)** The volcano plot of the differentially expressed genes on the endometrium of patients before and after PRP treatment groups. **(B)** Significantly enriched GO terms were selected based on a *P* < 0.05. Downregulated and upregulated GO terms are depicted in blue and red bars, respectively. **(C)** Boxplot of FPKM expression values for the twenty-two differentially expressed genes (*CD36*, *DUOX1*, *DUOX2*, *CLDN1*, *CLDN3*, *HPSE*, *KLF5*, *MET*, *TLR4*, *ARG2*, *LGALS9C*, *DEFB1*, *CCL3*, *CCL5*, *CCL21*, *CXCL12*, *CCR5*, *LYN*, *PIK3CG*, *RASGRP1*, *EPHB2*, and *EFNB1*) in RNA-sequencing. The y-axis represents the FPKM expression level. The color of the boxplot represents either the before-PRP treatment group (blue) or the after-PRP treatment group (red). CE, chronic endometritis; PRP, platelet-rich plasma; Go, Gene Ontology.

To unravel the modulatory mechanism of PRP at the molecular level, we conducted RNA sequencing analysis of the endometrial tissues of 3 cured persistent CE patients before (as control) and after PRP treatment. Principal component analysis for all samples and genes showed no obvious separation between the before and after PRP treatment groups (data not shown). To explore before and after PRP treatment changes, we performed differential expression gene (DEGs) analysis of endometrial transcriptional profiles with selection criteria of *P*-value < 0.05, and log2-transformed fold change value < 1, or > 1. We identified 669 upregulated and 700 downregulated genes (**Figure 5A**). Notably, GO analysis found that the upregulated DEGs were significantly enriched in the regulation of the endometrial receptivity and antimicrobial processes, such as positive regulation of wound healing, reactive oxygen species biosynthetic process, fatty acid transport, endothelial cell differentiation, epithelial cell development, antimicrobial humoral response, positive regulation of hemostasis, negative regulation of CD4^+^ αβ T cell proliferation, and toll-like receptor signaling pathway. In contrast, downregulated DEGs were enriched in immune response processes including natural killer cell chemotaxis, T cell chemotaxis, leukocyte chemotaxis, positive regulation of lymphocyte activation, antiviral innate immune response, lymphocyte proliferation, chemokine-mediated signaling pathway and leukocyte mediated immunity (**Figure 5B**). Interestingly, we also identified 22 candidate genes of immune cell-related and endometrial receptivity-related, including 10 downregulated genes (*CCL3*, *CCL5*, *CCL21*, *CXCL12*, *CCR5*, *LYN*, *PIK3CG*, *RASGRP1*, *EPHB2*, and *EFNB1*) and 12 upregulated genes (*CD36*, *DUOX1*, *DUOX2*, *CLDN1*, *CLDN3*, *HPSE*, *KLF5*, *MET*, *TLR4*, *ARG2*, *LGALS9C*, and *DEFB1*) (**Figure 5C**), implying that these genes might be the most critical changes before and after PRP treatment.

## Discussion

In this study, we sought to determine the effect of autologous intrauterine PRP infusion on persistent CE patients to understand its potential value for clinical applications. Here, the first major finding of our study was the high cure rate in persistent CE patients after PRP treatment. Second, the implantation rate and clinical pregnancy rate were significantly increased in the cured CE patients after PRP treatment, though no significant differences in pregnancy outcomes were observed between the PRP group and the control group. Third, our findings have indicated significantly decreased proportions of endometrial CD56^+^ NK cells, CD8^+^ T cells, Fopx3^+^ Treg cells, and T-bet^+^ Th1 cells in persistent CE patients after PRP treatment. Fourth, the transcriptome profile in endometrial confirmed that PRP treatment has unique immunomodulatory effects on the endometrium of patients with persistent CE. Taken together, autologous PRP treatment contributes to endometrial receptivity by reconstructing the local immune microenvironment in the uterus of patients with persistent CE.

In recent years, increasing evidence has shown that autologous PRP treatment plays a positive role in endometrium disorders, including thin endometrium (18), intrauterine adhesion (19), and recurrent implantation failure (RIF) (20). With the intrauterine infusion of PRP, numerous proteins, growth factors, and cytokines stored in the platelet interact with the endometrium through the promotion of cell proliferation and angiogenesis, and anti-inflammatory properties, resulting in successful implantation (21). Nevertheless, the available facts concerning the efficacy of PRP in persistent CE are limited. Although a recent study investigated the effects of antibiotics combined with PRP therapy on pregnancy outcomes following FET in RIF patients with CE (14), our research highlighted the efficacy of PRP monotherapy on the expression of endometrial CD138^+^ plasma cells and the pregnancy outcomes and further unraveled the underlying regulatory mechanisms of PRP in the endometrium of patients with persistent CE. The result of immunohistochemical staining for CD138^+^ plasma cells showed that 60.6% of patients converted to CE negative after PRP treatment, suggesting that the PRP treatment plays a positive role in treating persistent CE.

It is worth noting that antibiotics can exhibit tissue retention. It is important to consider whether the effectiveness of PRP monotherapy in reducing the presence of endometrial CD138^+^ plasma cells is due to the retention of antibiotics in endometrial tissue. In this study, patients with persistent CE underwent two rounds of antibiotic treatment. An endometrial biopsy was conducted after 7 days of each treatment. Previous studies have shown that the half-life of doxycycline, metronidazole, and levofloxacin in tissues is 18–22 hours (22, 23), 6–10 hours (24), and 7–8 hours (25), respectively. It is important to note that patients received PRP treatment on days 8–9 of the following menstrual cycle after completing two rounds of antibiotic treatment. This means that the first PRP treatment was approximately 3 weeks after the end of the antibiotic treatment. Furthermore, the endometrial biopsy after PRP treatment was performed 1 month after the last endometrial biopsy. Therefore, it can be concluded that the therapeutic effect of PRP on persistent CE is not affected by the retention of antibiotics in the tissue.

CE is a poorly investigated pathology that has been related to adverse reproductive outcomes, such as RIF and recurrent miscarriage (RM) (26, 27). In our previous study, we found a significantly reduced clinical pregnancy rate in RIF patients with CE compared with RIF women without CE (20.0% *vs.* 46.9%, *P* = 0.04) (28). It has been reported that CE affected embryo implantation by altering endometrial receptivity (29). The researchers considered that an abnormal number of plasma cells can be diagnosed as CE, which negatively affects embryo implantation. The mechanism behind this is believed to be related to the presence of microbes in the uterine cavity. These microbes release pathogenic agents, which can cause abnormal levels of immune cells and the expression of chemokines. This disruption of the endometrial microenvironment reduces its receptivity, ultimately leading to the failure of embryo implantation (30). In the present study, although we found no statistically significant difference in the clinical pregnancy rate between the control group and PRP group, there was a trend toward an increased clinical pregnancy rate in persistent CE women with PRP treatment when compared to RIF women with non-CE (57.6% *vs.* 46.9%) and RIF women with CE (57.6% *vs.* 20.0%), respectively. Moreover, the implantation rate and clinical pregnancy rate were significantly increased in the cured CE patients compared with non-cured CE patients after PRP treatment. Our data is consistent with a previous study, which has shown that PRP can potentially improve pregnancy outcomes in women with CE (6). Based on these results, we assume that PRP treatment may improve pregnancy outcomes by restoring the endometrial receptivity of women with persistent CE.

Endometrial physiology relies on a dynamic cell-to-cell dialogue between the stroma and epithelium compartments with a mixture of vascular and immune cells (31). The immune environment of the endometrium is closely related to endometrial receptivity (32). Several theories have been proposed to explain the impaired endometrial receptivity associated with CE, including the activation of local inflammatory processes, resulting in altered cytokine and chemokine secretion (33), abnormal infiltration of leukocytes within the endometrium (15), dissociated maturation between epithelial cells and stromal fibroblasts (34), defective decidualization (35), and defective endometrial vascularization (36). Notably, our previous study found a significantly high increase in the proportions of endometrial CD8^+^ T cells and Foxp3^+^ Treg cells in CE patients with RIF (15). Meanwhile, another study also reported that the uterine NK cell density in RM women with CE was significantly higher than those without CE (37). These studies indicated that CE is related to the changes in the endometrial immune microenvironment in patients with recurrent reproductive failure. To better understand the mechanisms underlying the immunoregulatory properties of PRP, we investigated whether the uterine infusion of PRP could modulate the local inflammatory response and modify the intrauterine transcriptomic profiles in patients with persistent CE after PRP treatment. Our analysis therefore shows that the proportions of endometrial CD56^+^ NK cells, CD8^+^ T cells, Foxp3^+^ Treg cells, and T-bet^+^ Th1 cells were significantly decreased in persistent CE patients after PRP treatment compared with those before PRP treatment. Moreover, we verified that the proportions of these endometrial immune cells were also specifically significantly decreased in patients with cured CE compared to those of non-cured CE patients after PRP treatment. This result indicated that the modulation of the endometrial immune cells by autologous PRP treatment appeared to be an important mechanism by which it improves endometrial receptivity.

In addition, we conducted a transcriptome analysis to explore changes in the endometrium before and after PRP treatment. Our results suggested that endometrial receptivity appears to be improved after PRP treatment, as reflected in the expression patterns of endometrial receptivity-related genes *CD36*, *DUOX1*, *DUOX2*, *CLDN1*, *CLDN3*, *HPSE*, *KLF5*, *MET*, *TLR4*, *ARG2*, *LGALS9C*, and *DEFB1* were significantly upregulated following PRP treatment. The transition into the receptive phase of the endometrium occurs with an abrupt transcriptomic inhibition in the immune-related process to reach a state where immune cell chemotaxis- and activation-associated genes, such as *CCL3*, *CCL5*, *CCL21*, *CXCL21*, *CCR5*, *LYN*, *PI3KCG*, *RASGRP1*, *EPHB2*, and *EFNB1* were uniformly and highly downregulated. A recent study identified that 12 immunoglobulin-related genes (*IGKC*, *IGHG1*, *IGHG4*, *IGLC3*, *IGHG3*, *IGLC2*, *IGHA1*, *IGKV3-20*, *IGLC1*, *IGHG*2, *JCHAIN*, and *IGHA2*) were upregulated in the CE endometria (38). However, no significant difference in these immunoglobulin-related genes was observed in the endometrium from patients before and after PRP treatment. Previous animal experiments showed that PRP treatment decreased the expression of inflammatory markers and fibrosis, increased the endometrial proliferation rate, and increased the proliferation gene expression (39). The enrichment analysis of DEGs before and after PRP treatment indicated that the most important effect of PRP treatment on the endometrium was to adjust the immune environment and promote tissue hemostasis of the endometrium. We provide a cross-talk molecular characterization of PRP treatment for improving endometrial receptivity, which can inform future studies.

PRP treatment has achieved encouraging results in clinical practice. By comparing the endometrium of the same patient before and after PRP treatment, the possible mechanism of PRP treatment to improve endometrial receptivity was described. However, this study still had limitations. Firstly, although we used the number of CD138^+^ cells in the endometrial stroma to provide clinically relevant diagnostic criteria for CE (2), we must acknowledge that optimal threshold values for sensitivity and specificity are not well defined by the CD138^+^ immunohistochemical staining method to stain plasma cells to diagnose CE (40). Secondly, regarding the experiment on the effect of PRP treatment on endometrial immunological response, the number of patients was 33; thus, the sample size was small to evaluate the effect of PRP treatment correctly. Moreover, the control group selected patients without CE rather than patients with persistent CE without PRP treatment, which did not directly demonstrate the therapeutic effect of PRP on patients with persistent CE. Thus, more powerful studies with a much larger sample size and an ideal control group are needed to elucidate the underlying effect. Thirdly, more abundant immune cell information could not be obtained. This is what we will overcome in further research. Fourthly, although the RNA-seq results provide preliminary mechanistic insights, future studies with a larger sample size are needed to validate the uterine-specific role of PRP in patients with persistent CE. Overall, using PRP may help ensure a better cured rate, favorable pregnancy outcomes, and optimal endometrial receptivity in our study. We found that the critical mechanism by which PRP treatment improves endometrial receptivity lies in the modulation of the endometrial immune microenvironment. Our findings provide evidence underscoring the essential role of autologous PRP as an alternative therapeutic tool for persistent CE. Verifying our findings in larger patient groups through randomized controlled studies would strengthen this finding and secure the role of PRP as a successful therapeutic means for patients with persistent CE, especially for those who fail to respond to conventional antibiotic schemes.

## Data Availability

The datasets presented in this study can be found in online repositories. The names of the repository/repositories and accession number(s) can be found in the article/**Supplementary Material**.
