# Supplementary material for: Intrauterine infusion of autologous platelet-rich plasma modulates endometrial immune status and improves pregnancy outcomes in patients with persistent chronic endometritis
Source: Front Immunol. 2025 May 15;16:1528522. doi: 10.3389/fimmu.2025.1528522 (PMC12119290; doi:10.3389/fimmu.2025.1528522)
Supplement: Supplementary file 1 [file Table1.docx]

**Table S1.** Details of sources and concentrations of antibodies used for immunohistochemistry staining

| **Antibody** | **Manufacturer** | **Catalog number** | **Dilution** |
| --- | --- | --- | --- |
| Anti-CD138 | Gene Tech | GT212629 | 1:100 |
| Anti-CD8 | Novocastra | NCL-L-CD8-4B11 | 1:150 |
| Anti-CD56 | Gene Tech | GT200529 | 1:200 |
| Anti-CD68 | Novocastra | NCL-CD68-KP1 | 1:100 |
| Anti-CD163 | Novocastra | NCL-L-CD163 | 1:1200 |
| Anti-Foxp3 | eBioscience | 14-4777 | 1:100 |
| Anti-Tet | BD Biosciences | 561265 | 1:800 |
| Anti-GATA3 | R & D Systems | MAB6330 | 1:2000 |
